# Clinical outcomes of COVID-19 infection in liver transplant recipients based on vaccination status

**DOI:** 10.3389/frtra.2024.1515964

**Published:** 2025-01-09

**Authors:** Vinathi Polamraju, Neeta Vachharajani, Brian F. Gage, Jeffrey S. Crippin, William C. Chapman

**Affiliations:** ^1^ Department of Medicine, Washington University School of Medicine, St. Louis, MO, United States; ^2^ Section of Transplant Surgery, Washington University School of Medicine, St. Louis, MO, United States

**Keywords:** liver transplantation, COVID-19, vaccination, disease severity, risk factors

## Abstract

**Background:**

COVID-19 disease burden has been mitigated by vaccination; however, concerns persist regarding weakened immune responses in liver transplant (LT) recipients. This study investigates COVID-19 outcomes in LT recipients based on vaccination status.

**Methods:**

This single-center retrospective study identified LT recipients with PCR-confirmed COVID-19 infection from 03/01/2020 to 07/31/2023. Logistic regression analyses were conducted, adjusting for age, race, co-morbidities, number of immunosuppressive agents, and infection date.

**Results:**

Of 1,787 registered LT recipients, 361 had confirmed COVID-19 infection. Of those, 136 were unvaccinated and 225 were vaccinated. 13% had 1 vaccine dose, 31% had 2 vaccine doses, and 56% had 3 vaccine doses prior to infection. Logistic regression found higher mortality (*p* = 0.001) and hospitalization (*p* = 0.016) rates for older recipients, while those with 3 or more vaccine doses had lower mortality (*p* = 0.039) and hospitalization (*p* = 0.008) rates. Chronic kidney disease (CKD) increased risk of hospitalization (*p* < 0.001). Adjusting for the date when the Omicron variant became locally predominant, the protective effect from 3 or more vaccine doses declined to an OR (95% CI) of 0.58 (0.15–2.23), *p* = 0.39.

**Conclusions:**

Three or more COVID-19 vaccine doses could decrease mortality for LT recipients, particularly older recipients and those with CKD. These individuals may benefit from vaccination and other interventions.

## Introduction

COVID-19 had devastating effects worldwide with high rates of disease burden in both developing and developed countries (1, 2). In the United States also, there have been over 90 million cases confirmed with an approximate 1.1% case fatality rate (3). The immune dysregulation and clinical sequelae of COVID-19 infection were promptly characterized (4, 5), but much has changed since the advent of vaccination. Vaccination against COVID-19 decreases mortality, hospitalizations, and severity of infection (6–8). However, early vaccine trials excluded immunocompromised recipients (9–11), and there are conflicting and limited data regarding vaccine efficacy amongst liver transplant (LT) recipients (12–14). LT recipients take immunosuppressants and have co-morbidities that predispose them to COVID-19 infection (15–17). Notably, they have suboptimal seroconversion rates following COVID-19 vaccines ranging from 22.4% after one dose to greater than 90% following three doses (13, 18–20). However, it has yet to be determined whether this lower antibody response correlates with disease outcome and severity. Some studies suggest that LT recipients, both vaccinated and unvaccinated, are at an increased risk of contracting COVID-19 infection but not necessarily of COVID-19-associated mortality (17, 21–23). Understanding disease severity and clinical outcomes of COVID-19 infection in vaccinated LT recipients compared to their unvaccinated counterparts will help uncover several crucial and widely understudied questions. These include but are not limited to, evaluating the clinical efficacy of vaccines in the background of various medical co-morbidities for LT recipients, directing vaccine outreach towards specific vulnerable LT recipients, highlighting the need for targeted treatment strategies to mitigate adverse outcomes among hospitalized recipients, directing further research to enhance understanding of COVID-19 infection on liver transplant recipients, etc. Overall, our aim was to contribute additional insights to the limited literature regarding the impact of COVID-19 infection for liver transplant recipients based on their vaccination status.

## Materials & Methods

This was a retrospective cohort study conducted at a tertiary high-volume transplant center. Waiver of consent was justified due to the use of retrospective, de-identified recipient information. This study was approved by the institutional review board of the Washington University School of Medicine and research reported was supported in part by the Washington University Institute of Clinical and Translational Sciences grant UL1TR002345 from the National Center for Advancing Translational Sciences (NCATS) of the NIH. A prospectively maintained LT database was used to identify adult LT recipients with laboratory-confirmed COVID-19 infection from 03/01/2020 to 07/31/2023. Laboratory-confirmed infection was defined by positive testing via nucleic acid amplification testing or antigen testing. Those included were patients greater than the age of 18 years. To establish a cohort of hospitalized patients, we utilized International Classification of Diseases 10th Edition (ICD-10) codes in conjunction with patient chart reviews. First, we reviewed each patient's medical chart to identify encounters with positive COVID-19 testing as defined above. After this initial screen, we analyzed the primary position ICD-10 codes to determine the primary reason for hospitalization.

Patient demographics, comorbidities including indications for liver transplantation, medications including immunosuppressive medications, vaccinations (number and type), hospitalizations, and major outcomes were extracted via a single electronic medical record system. For patients who had >1 hospitalization for COVID, we studied the 1st admission.

Descriptive analyses were performed to characterize the overall cohort, hospitalized cohort (Supplementary Table S1), and outcomes amongst hospitalized patients. Unless otherwise specified, results are expressed as a median and interquartile range or counts and percentages. Univariate logistic regression analyses were performed to identify risk factors for hospitalization and mortality amongst COVID-19 infected LT recipients (Supplementary Table S2). Multivariable logistic regression analyses were then performed, adjusting for covariates including age, race, co-morbidities (diabetes, CKD, HTN), vaccination status, and number/type of immunosuppressive agents. Similar analysis was run to predict factors associated with mortality amongst COVID-19 infected LT recipients, adjusting for the date (12/20/2021) when the omicron variant became prevalent in our population (24). Due to the limited outcome variables available in this single-center retrospective analysis, additional co-variate adjustments were deferred. Statistical analyses were performed using GraphPad Prism v 10.0.1 (GraphPad Software, Inc. Boston, MA), SAS version 9.4 (SAS Institute Inc., Cary, NS), and SPSS version 26 (IBM SPSS Statistics, IBM Corporation, Armonk, NY). Categorical variables were compared using Fisher's exact test or Chi-Square test as applicable. Mann-Whitney *U* test was used to compare continuous variables. Survival graphs were plotted using Kaplan-Meier curves, and survival rates were compared using the log-rank test. The outcome variables of interest remained mortality and hospitalization rates. For every comparison, a two-sided *p*-value <0.05 was considered statistically significant.

## Results

361 out of 1,787 registered LT recipients had PCR-confirmed COVID-19 infection from 03/01/2020 to 07/31/2023. 136 COVID-19 infected LT recipients were unvaccinated at the time of infection and 225 were vaccinated. Of these 225, about 13% had 1 vaccine dose, 31% had 2 vaccine doses, and 56% had 3 or more vaccine doses prior to COVID-19 infection. Roughly 93% of our cohort received mRNA-based vaccinations and the remainder received viral vector vaccinations. In our cohort, 89.2% of patients were Caucasian. The average age was 64 years with about an equal number of male and female patients. Most individuals were overweight (i.e., BMI >28 kg/m^2^). With regards to co-morbidities, 37% had diabetes, 60% had hypertension, and 47% had chronic kidney disease (i.e., eGFR <60 ml/min/1.73 m^2^). Only 16% of recipients were within two years post liver transplantation. The most common reasons for transplantation were hepatocellular carcinoma, alcoholic cirrhosis, and NASH cirrhosis. Most (64%) patients were on dual- or triple-immunosuppressants at the time of infection (Table 1).

**Table 1:** Baseline patient demographics.

	Overall (*n* = 361)	Vaccinated (*n* = 225)	Unvaccinated (*n* = 136)	*p* value
Age, median (IQR)	64 (52–71)	66 (57–72)	60 (42–69)	<0.0001
Sex				0.045
Female	151 (41.8%)	85 (37.8%)	66 (48.5%)	
Male	210 (58.2%)	140 (62.2%)	70 (51.5%)	
Race				0.753
African American	33 (9.1%)	17 (7.6%)	16 (11.8%)	
Caucasian	322 (89.2%)	204 (90.7%)	118 (86.8%)	
Other	6 (1.7%)	4 (1.8%)	2 (1.5%)	
BMI (kg/m^2^)	28.3 (25–33)	29 (25–33)	28 (25–32)	0.104
Diabetes				
Yes	133 (36.8%)	85 (37.8%)	48 (35.3%)	0.225
Hypertension				
Yes	218 (60.4%)	139 (61.8%)	79 (58.1%)	0.487
CKD				
Yes	169 (46.8%)	114 (50.7%)	55 (40.4%)	0.059
Number of vaccine doses				
Unvaccinated		–	136 (100%)	
1		30 (13.3%)	–	
2		70 (31.1%)	–	
≥3		125 (55.6%)	–	
Vaccine type				
JNJ-78436735 (J&J)		14 (6.2%)	–	
BNT162b2 (Pfizer)		120 (53.3%)	–	
mRNA-1273 (Moderna)		91 (40.4%)	–	
<2 years post LT				
Yes	56 (15.5%)	38 (16.9%)	18 (13.2%)	0.353
Indication for LT				0.185
HCC	77 (21.3%)	49 (21.8%)	28 (20.6%)	
Alcoholic cirrhosis	66 (18.3%)	44 (19.6%)	22 (16.2%)	
NASH cirrhosis	68 (18.8%)	52 (23.1%)	16 (11.8%)	
Other	150 (41.6%)	80 (35.6%)	70 (51.4%)	
Immunosuppression				0.804
Azathioprine	14 (3.9%)	7 (3.1%)	7 (5.1%)	
Sirolimus	8 (2.2%)	5 (2.2%)	3 (2.2%)	
Everolimus	18 (5%)	13 (5.8%)	5 (3.7%)	
MMF	184 (51%)	109 (48.4%)	75 (55.1%)	
Cyclosporine	21 (5.8%)	12 (5.3%)	9 (6.6%)	
Tacrolimus	313 (86.7%)	200 (88.9%)	113 (83.1%)	
Corticosteroids	80 (22.2%)	51 (22.7%)	29 (21.3%)	
Number of IS therapies				0.202
0	8 (2.2%)	3 (1.3%)	5 (3.7%)	
1	123 (34.1%)	83 (36.9%)	40 (29.4%)	
2	175 (48.5%)	103 (45.8%)	72 (52.9%)	
3	55 (15.2%)	36 (16%)	19 (14%)	
Hospitalization				0.473
Yes	127 (35.2%)	76 (33.8%)	51 (37.5%)	
No	234 (64.8%)	149 (66.2%)	85 (62.5%)	
COVID-19 therapies				
Anti-viral				
Paxlovid	16 (4.4%)	12 (5.3%)	4 (2.9%)	0.290
Remdesivir	100 (27.7%)	58 (25.8%)	42 (30.9%)	0.297
Immunotherapy				
Monoclonal antibodies	119 (33%)	93 (41.3%)	26 (19.1%)	<0.0001
Convalescent plasma	9 (2.5%)	1 (0.4%)	8 (5.9%)	0.0013
Immunomodulatory therapy				
Baricitinib	2 (0.6%)	2 (0.9%)	0 (0%)	0.27
Steroids	82 (22.7%)	47 (20.9%)	35 (25.7%)	0.30
None	126 (34.9%)	62 (27.6%)	64 (47.1%)	0.068
Death				
30-day mortality	20 (5.5%)	12 (5.3%)	8 (5.9%)	0.817
60-day mortality	27 (7.5%)	15 (6.7%)	12 (8.8%)	0.536

BMI, body mass index; CKD, chronic kidney disease; HCC, hepatocellular carcinoma; IQR, interquartile range; IS, immunosuppression; LT, liver transplant; MMF, mycophenolate mofetil; NASH, non-alcoholic steatohepatitis.

Among the vaccinated cohort, 62.2% were male and 37.8% were female whereas in the unvaccinated cohort, 51.5% were male and 48.5% were female (*p* = 0.045). Also, vaccinated LT recipients were slightly older than their unvaccinated counterparts (66 years vs. 60 years, *p* < 0.0001). Other recipient-specific factors were comparable between the unvaccinated and vaccinated cohorts (Table 1).

Overall, 127 recipients were hospitalized at the time of COVID-19 diagnosis. Most (65%) were hospitalized for pneumonia due to COVID-19 (J12.82), 28% were hospitalized for COVID-19 related liver injury (T86.49), 7% were asymptomatic but incidentally noted to be COVID-19 positive on admission for alternative reasons. Again, the vaccinated cohort was slightly older than their unvaccinated counterparts (66 years vs. 60 years, *p* = 0.012). All other factors were comparable between the unvaccinated and vaccinated hospitalized patients. 20 patients died within 30 days of positive COVID-19 test results and 27 patients died within 60 days of positive COVID-19 test results (Supplementary Table S1). Data on the use of COVID-19 specific therapies were considered to assess their impact on clinical outcomes. In the total cohort of 361 COVID-19 positive LT recipients, 126 recipients did not receive any specific therapies. The remaining 235 recipients received the following treatments: 6.8% received Paxlovid, 42.6% received Remdesivir, 50.6% received monoclonal antibodies, 3.8% received convalescent plasma, 0.9% received baricitinib, 34.9% received steroids at a higher dose than their baseline immunosuppression (either intravenous dexamethasone or oral prednisone), and 4.7% received two different forms of COVID-19 therapies (Table 1).

Univariate analysis conducted on the full (unmatched) dataset noted no significant differences in mortality or hospitalization based on vaccination status (Supplementary Table S2). However, on multivariable analysis (Table 2), older age was associated with an increased mortality [OR per decade 2.30 (95% CI 1.415–3.752), *p* = 0.001] and hospitalization [OR 1.02 (95% CI 1.004–1.040), *p* = 0.016]. Having 3 or more vaccine doses at the time of infection was associated with reduced mortality [OR 0.28 (95% CI 0.097–0.805), *p* = 0.039] and hospitalization [OR 0.47 (95% CI 0.268–0.821), *p* = 0.008]. Chronic kidney disease (CKD) was associated with an increased risk of hospitalization [OR 2.34 (95% CI 1.442–3.790), *p* < 0.001] but not mortality. Number of immunosuppressive therapies at the time of infection did not increase risks of mortality or hospitalization.

**Table 2:** Multivariate logistic regression analysis of factors associated with mortality and hospitalization amongst COVID-19 positive liver transplant patients.

	COVID-19 mortality (*n* = 43)		COVID-19 hospitalization (*n* = 127)	
	OR (95% CI)	*p* value	OR (95% CI)	*p* value
Age	2.304 (1.415–3.752)	0.001	1.022 (1.004, 1.040)	0.016
Gender				
Female	(Reference)		(Reference)	
Male	0.981 (0.499, 1.931)	0.957	0.864 (0.545, 1.369)	0.533
Co-morbidities				
Diabetes	2.247 (0.958, 5.271)	0.063	1.533 (0.963, 2.440)	0.072
Hypertension	1.078 (0.515, 2.258)	0.842	0.720 (0.437, 1.185)	0.196
CKD	2.517 (0.991, 6.395)	0.052	2.337 (1.442, 3.790)	<0.001
Vaccination status				
Unvaccinated	(Reference)		(Reference)	
1–2 vaccine doses	0.622 (0.228, 1.694)	0.732	0.849 (0.484, 1.491)	0.569
3 or more vaccine doses	0.279 (0.097, 0.805)	0.039	0.469 (0.268, 0.821)	0.008
Number of IS therapies	1.305 (0.685–2.486)	0.421	1.201 (0.387, 3.143)	0.562

CKD, chronic kidney disease; IS, immunosuppressive therapies.

When adjusted for the date that Omicron became the predominant strain (Table 3), older age [OR per decade 2.26 (95% CI 1.381–3.685), *p* = 0.001] and CKD [OR 2.67 (95% CI 1.039–6.872), *p* = 0.041] had increased mortality. LT recipients infected after the Omicron variant became strain had lower mortality, albeit not statistically significant [OR 0.37 (95% CI 0.123–1.106), *p* = 0.075]. Those with 3 or more vaccine doses prior to infection also had lower odds of mortality when compared to those with fewer vaccinations, albeit not statistically significant [OR 0.58 (95% CI 0.148–2.229), *p* = 0.390]. Number of immunosuppressive therapies at the time of infection again did not increase mortality.

**Table 3:** Multivariate logistic regression analysis of factors associated with mortality amongst COVID-19 positive liver transplant patients, adjusted for the Omicron variant.

	COVID-19 mortality (*n* = 43)	
	OR (95% CI)	*p* value
Age, per decade	2.256 (1.381, 3.685)	0.001
Race		
African American	1.222 (0.352, 4.240)	0.750
Co-morbidities		
Diabetes	2.116 (0.891, 5.025)	0.090
Hypertension	1.892 (0.372, 3.574)	0.102
CKD	2.673 (1.039, 6.872)	0.041
Vaccination status		
Unvaccinated	(Reference)	
1–2 vaccine doses	0.924 (0.304, 2.808)	0.690
3 or more vaccine doses	0.575 (0.148, 2.229)	0.390
Number of IS therapies	1.269 (0.665, 2.424)	0.470
Omicron variant	0.369 (0.123, 1.106)	0.075

CKD, chronic kidney disease; IS, immunosuppressive therapies.

No significant differences in length of hospital stay, length of ICU stay, need for mechanical ventilation or dialysis, need for supplemental oxygen during or after hospitalization were noted. However, hospitalized LT recipients with 3 or more vaccines doses prior to infection had lower rates of ICU admission when compared to those with 2 or fewer vaccine doses (13.9% vs. 31.9%, *p* = 0.039). 96% of hospitalized LT recipients received anti-viral therapies during admission (Table 4).

**Table 4:** Outcomes amongst hospitalized patients.

	2 or fewer vaccine doses (*n* = 91)	3 or more vaccine doses (*n* = 36)	*p* value
Length of hospital stay (median, IQR)	8 days (1–43)	6 days (1–26)	0.478
Need for ICU admission (*n*, %)	29 (31.9%)	5 (13.9%)	0.039
Length of ICU stay (median, IQR)	7 days (3–23)	6 days (2–16)	0.692
Need for MV (*n*, %)	16 (17.6%)	2 (5.6%)	0.080
Need for dialysis	22 (24.2%)	8 (22.2%)	0.815
Need for supplemental oxygen during hospitalization	61 (67%)	22 (61.1%)	0.527
Need for supplemental oxygen after discharge	13 (14.3%)	2 (5.6%)	0.169
30-day mortality (*n*, %)	15 (16.5%)	4 (11.1%)	0.444

ICU, intensive care unit; IQR, interquartile range; MV, mechanical ventilation.

On multivariable analysis of vaccinated LT recipients, older age was again associated with increased risk of mortality [OR per decade 1.05 (95% CI 1.003–1.107), *p* = 0.038] and having received 3 or more vaccine doses was associated with reduced mortality [OR 0.32 (95% CI 0.116–0.904), *p* = 0.031]. However, older age, having 3 or more vaccine doses, and co-morbid CKD did not impact risk of hospitalization (Table 5).

**Table 5:** Factors associated with mortality and hospitalization amongst vaccinated COVID-19 positive liver transplant patients.

	COVID-19 mortality (*n* = 24)		COVID-19 hospitalization (*n* = 76)	
	OR (95% CI)	*p* value	OR (95% CI)	*p* value
Age	1.054 (1.003, 1.107)	0.038	1.023 (0.996, 1.051)	0.102
Gender				
Female	(Reference)		(Reference)	
Male	1.087 (0.393, 3.006)	0.872	1.201 (0.640, 2.245)	0.568
Co-morbidities				
Diabetes	2.913 (0.825, 1.038)	0.097	1.891 (0.998, 3.572)	0.056
Hypertension	1.444 (0.474, 4.403)	0.518	1.279 (0.503, 3.254)	0.606
CKD	2.366 (0.951, 5.887)	0.064	1.239 (0.639, 2.401)	0.526
Vaccination status				
1–2 vaccine doses	(Reference)		(Reference)	
3 or more vaccine doses	0.324 (0.116–0.904)	0.031	0.594 (0.318, 1.110)	0.103
IS regimen				
Prednisone therapy prior to COVID-19 infection	1.205 (0.019, 3.263)	0.196	1.053 (0.435, 2.553)	0.909
MMF only	1.970 (0.273, 1.42)	0.502	1.062 (0.372, 3030)	0.911
MMF + Tacrolimus	0.412 (0.291, 1.99)	0.414	0.896 (0.351, 2.286)	0.818

CKD, chronic kidney disease; IS, immunosuppressive.

**Figure 1:**
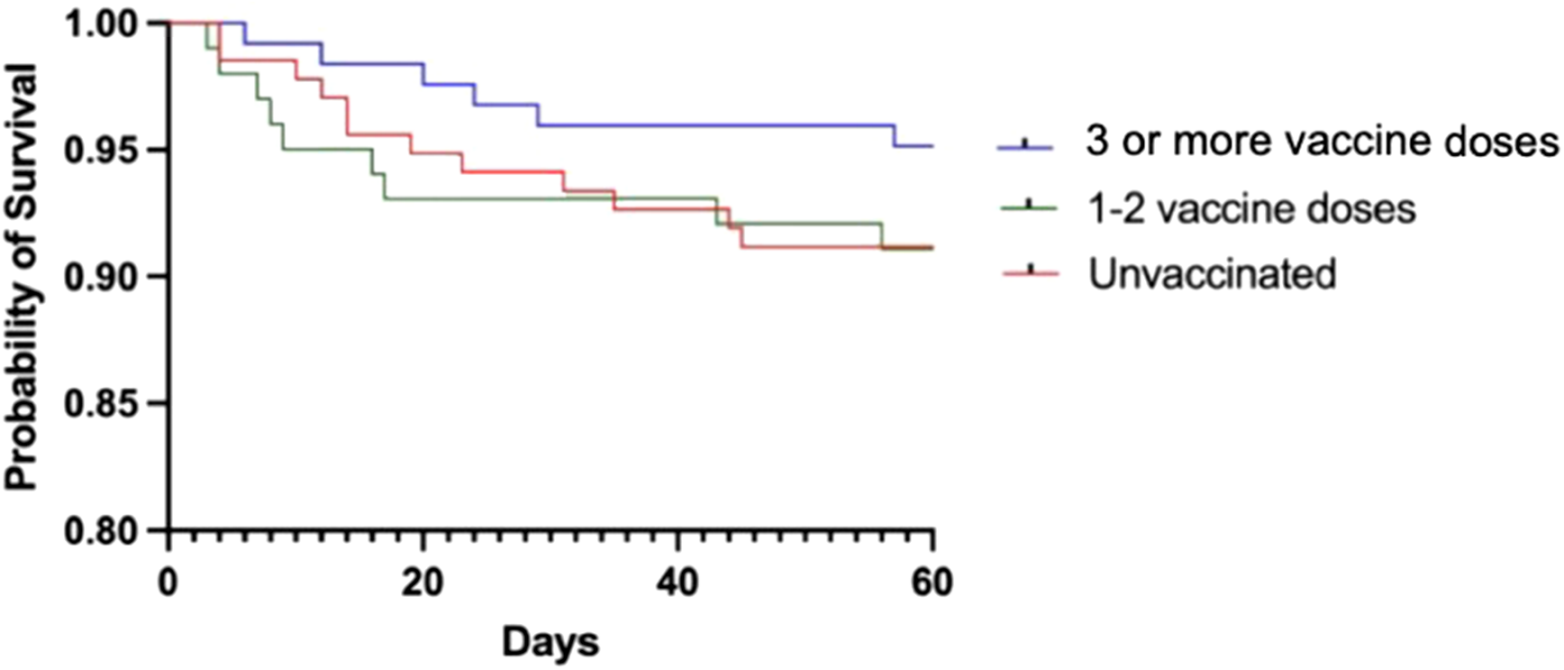
60-day survival outcomes from COVID-19 infection amongst all liver transplant patients. Levels of significance: *p* = 0.007.

Kaplan-Meier curves noted higher 60-day survival rates among recipients who received 3 or more vaccine doses (*p* = 0.007) (Figure 1). This finding was validated on a subset analysis focused only on vaccinated COVID-19 infected LT recipients. As shown in Supplementary Figure S1, cumulative survival rates amongst vaccinated LT recipients with 3 or more vaccine doses was higher when compared to those with two or fewer vaccine doses (*p* = 0.018).

## Discussion

Vaccination against COVID-19 is associated with less severe disease outcomes among infected LT recipients. This study has three key findings. First, receiving three or more COVID-19 vaccines prior to infection is associated with reduced mortality rates amongst liver transplant (LT) recipients. Second, older age and CKD increase the likelihood of hospitalization amongst COVID-19 infected LT recipients. However, this risk may be mitigated by vaccination. Last, among hospitalized COVID-19 infected LT recipients, ICU admission rates are lower among those with 3 or more vaccine doses prior to infection.

The reduced mortality noted among COVID-19 infected LT recipients with three or more vaccine doses is consistent with prior studies that note suboptimal antibody titers among LT recipients with only one or two doses that increases substantially following the third vaccine dose (25–28). Consistent with prior research (29), Omicron-infected LT recipients in this study tended to have lower mortality rates than recipients infected with earlier strains. Interestingly, when accounting for the date that Omicron became the local predominant strain, relative protection from vaccination was not statistically significant. The lack of statistical significance in these results may reflect multiple factors, including sample size limitations and potential confounders. These confounders include lower vaccine uptake in the post-Omicron era, immunity from potential prior infections, overall increased vaccination rates among the local population, changes in treatment protocols, and other shifts in virus variants. While these were not assessed in our study, further research is warranted. Importantly, it has been previously established that COVID-19 reinfection portends better clinical outcomes among solid-organ transplant recipients which could also explain the observed effect in this study (30).

During our study period, LT recipients received standard FDA-approved anti-viral therapies, including Paxlovid and Remdesivir, which have been shown to reduce viral load and improve outcomes in higher risk populations (31). While we did not monitor immunosuppressive dose adjustments, current literature suggests Paxlovid can increase calcineurin inhibitor levels (i.e., tacrolimus), requiring dose reduction, and MMF may heighten viral replication risk, often prompting temporary discontinuation (32). Steroids were also escalated in our study to control inflammation and mitigate risk of severe cytokine release (31, 32). Although the total number of immunosuppressive agents did not influence mortality for LT recipients in our study, even when adjusted for the Omicron variant, further studies are warranted to explore the correlation between mortality and changes in immunosuppressive therapies.

Evolving clinical practices, including introduction of new treatments, likely also contributed to improved outcomes in our study. Remdesivir, granted Emergency Use Authorization (EUA) by the FDA in May of 2020 and fully approved in October of 2020, dexamethasone recommended by the WHO in September of 2020 after positive RECOVERY trial results, and monoclonal antibodies, granted EUA in November of 2020, were key treatments (31, 32). They became increasingly utilized especially as Omicron became the predominant local strain in December of 2021. The increased availability and use of these therapies likely had a significant impact on patient outcomes, potentially improving results irrespective of vaccination status. Consequently, the lack of clear mortality benefit from vaccination in our study after adjusting for Omicron may reflect the greater influence of improved clinical management and treatment protocols rather than the vaccine itself.

In keeping with prior studies, this study also demonstrates that CKD and older age (among the total cohort and vaccinated subset) are associated with increased mortality and hospitalization amongst infected LT recipients (31–34). The impact of vaccination may be attenuated in this subgroup due to multiple factors like impaired immune responses and the presence of complex comordbities. Those with CKD, particularly those on dialysis, may have an impaired immune response. According to Toniutto et al., lower antibody titers following COVID-19 mRNA vaccines among LT recipients with CKD appear to correlate with higher hospitalization rates (20). Furthermore, CKD often co-exists with other conditions, such as diabetes, hypertension, and other cardiovascular diseases, which may exacerbate the risk of severe clinical outcomes following COVID-19 infection. These conditions may also reduce the vaccine's protective effect. By preventing hospitalizations and ICU admission by vaccinating LT recipients, we may reduce hospitalizations and mortality. This underscores the need for increased attention to be directed towards COVID-19 infected liver transplant recipients who may be particularly vulnerable.

The observed reduction in ICU admission rates suggest that vaccination may help prevent severe disease, possibly by reducing progression to critical illness in high-risk patients. However, it did not significantly impact other outcomes such as length of stay or need for supplemental oxygen. This may be explained by the complex interplay of factors influencing those outcomes, including the presence of other severe pre-existing medical conditions. Additionally, the vaccine may be more effective in preventing the most severe stages of the illness than in altering the overall disease progression. However, further studies should explore whether vaccination has differential effects on these secondary outcomes among other subgroups of patients, such as those with different comorbidities or varying levels of disease severity. Uncovering these factors can clarify specific contexts in which vaccination provides the most benefit in reducing hospitalization related outcomes for LT recipients.

A strength of our study is that we analyzed patient data from both the pre- and post-vaccine eras, and as a result, we were able to capture the evolving COVID-19 landscape. Multiple variables were used to adjust for confounding factors, including date of infection. Additionally, we considered only LT recipients and excluded patients with indications for alternative immunosuppressive regimens. One limitation is that, as a single-center retrospective study, patient demographics may not be representative: specifically, our cohort was predominantly male and Caucasian, and they received mostly mRNA-based vaccines. This limits generalizability of our findings as other regions with differences in vaccine types, demographics, and clinical practices may have varying outcomes. Specifically, prior studies have suggested that race and gender may influence vaccine response. Women, particularly younger in age, typically have stronger immune responses to vaccines than men, as observed in vaccine trials for influenza, COVID-19, and others, which may be due to hormonal or genetic difference (35). This contrasts with males and racial groups, particularly African American and Hispanic populations, where vaccine hesitancy and lower uptake are more prevalent (36). The predominance of Caucasian males in our study could have impacted our results. Also, we missed individuals who were asymptomatic, did not pursue COVID-19 testing, or received care for COVID-19 outside of our network. While we only included data from each patient's first known COVID-19 infection, some patients may have had prior asymptomatic infections (or symptomatic infections treated outside of network). Moreover, exposure risk factors such as mask-wearing behaviors were not measured. Further differences in health behavior amongst vaccinated vs. unvaccinated individuals, changes in treatment medications, and provider skills were not captured.

Future multicenter, prospective studies are needed to determine the long-term efficacy of COVID-19 vaccines in maintaining immunogenicity on evolving variants. Further investigation is necessary to elucidate the number of vaccines and the interval duration between them in organ transplant recipients. Another area of interest lies in investigating the impact of COVID-19 vaccination for those on the transplant list as opposed to post-transplant recipients as analyzed in our study. Based on current evidence and observations from this study, vaccination strategies could benefit from prioritizing high-risk populations, namely those of older age and with co-morbid CKD. More frequent booster doses for those at elevated risk of severe disease or breakthrough infections could provide potential benefit. Tailoring vaccination schedules, including booster schedules, that account for a recipient's age, comorbidities, and transplant status could help optimize protection. Such individualized approaches can significantly impact public health strategies, particularly by improving vaccination coverage and booster uptake among LT recipients with medical comorbidities. The protection of COVID-19 vaccination should be highlighted: reduce costs, iatrogenic infections, ICU admissions, and mortality.

Additional supporting information may be found online in the Supporting Information section at the end of the article.

## Data Availability

The raw data supporting the conclusions of this article will be made available by the authors, without undue reservation.
