# Supplementary material for: Clinical outcomes of COVID-19 infection in liver transplant recipients based on vaccination status
Source: Front Transplant. 2025 Jan 9;3:1515964. doi: 10.3389/frtra.2024.1515964 (PMC11754219; doi:10.3389/frtra.2024.1515964)
Supplement: Supplementary file 1 [file Table1.pdf]

Table S1. Baseline demographics of hospitalized patients (BMI = body mass index, CKD = chronic kidney disease, HCC = hepatocellular carcinoma, IQR = interquartile range, IS = immunosuppression, LT = liver transplant, MMF = mycophenolate mofetil, NASH = non-alcoholic steatohepatitis)

	**Unvaccinated (n=53)**	**Vaccinated (n=74)**	**p value**
Age, mean (SD)	60 (14.5)	66 years (11.9)	0.012
Gender			
Male	26 (49.1%)	45 (60.8%)	0.096
Female	27 (50.9%)	29 (39.2%)	
Ethnicity			
Caucasian	42 (79.2%)	62 (83.8%)	0.402
African American	11 (20.8%)	10 (13.5%)	
Other	-	2 (2.7%)	
BMI, mean (SD)	28 (5.7)	28 (6.2)	1.001
Comorbidities			
T2DM	23 (43.4%)	35 (47.3%)	0.581
HTN	32 (60.4%)	46 (62.2%)	0.794
CKD	35 (66.0%)	43 (58.1%)	0.250
Number of vaccine doses			
1	-	11 (14.9%)	
2	-	27 (36.5%)	
≥3	-	36 (48.6%)	
Vaccine type			
JNJ-78436735 (J&J)	-	5 (6.8%)	
BNT162b2 (Pfizer)	-	43 (58.1%)	
mRNA-1273 (Moderna)	-	26 (35.1%)	
<2 years post LT			
Yes	9 (17%)	21 (28.4%)	0.136
Immunosuppression			0.936
Azathioprine	2 (3.8%)	3 (4.1%)	
Sirolimus	1 (1.9%)	1 (1.4%)	
Everolimus	1 (1.9%)	4 (5.4%)	
MMF	33 (62.3%)	37 (50%)	
Cyclosporine	6 (11.3%)	4 (5.4%)	
Tacrolimus	41 (77.4%)	63 (85.1%)	
Corticosteroids	13 (24.5%)	16 (21.6%)	
Number of IS therapies			0.94
0	2 (3.8%)	3 (4.1%)	
1	14 (26.4%)	26 (35.1%)	

2	28 (52.8%)	33 (44.6%)	
3	9 (17%)	12 (16.2%)	
