# Supplementary material for: Clinical outcomes of COVID-19 infection in liver transplant recipients based on vaccination status
Source: Front Transplant. 2025 Jan 9;3:1515964. doi: 10.3389/frtra.2024.1515964 (PMC11754219; doi:10.3389/frtra.2024.1515964)
Supplement: Supplementary file 2 [file Table2.pdf]

Table S2. Univariate analysis of factors associated with mortality and hospitalization amongst COVID-19 positive liver transplant patients (CKD = chronic kidney disease)

	**COVID-19 Mortality (n=43)**		**COVID-19 Hospitalization (n=127)**	
	OR (95% CI)	p value	OR (95% CI)	p value
Age (years)	1.043 (1.010, 1.077)	0.010	3.816 (0.085, 7.547)	0.045
Gender				
Female	(Reference)		(Reference)	
Male	1.015 (0.508, 2.028)	0.966	0.941 (0.524, 1.661)	0.884
Co-morbidities				
Diabetes	1.141 (0.530-2.458)	0.736	1.813 (0.945, 3.141)	0.062
Hypertension	1.158 (0.350-2.625)	0.934	1.033 (0.580, 1.863)	1.000
CKD	2.564 (0.245-2.69)	0.432	1.479 (0.840, 2.544)	0.202
Vaccination Status				
Unvaccinated	(Reference)		(Reference)	
1-2 vaccine doses	0.971 (0.448-2.105)	0.941	0.849 (0.484, 1.491)	0.569
3 or more vaccine doses	0.774 (0.997-6.645)	0.051	0.921 (0.516-1.645)	0.781
Immunosuppressive regimen				
Prednisone therapy prior to COVID-19 infection	1.707 (0.253-3.976)	0.508	1.074 (0.563-3.048)	0.829
MMF only	2.737 (0.277-5.960)	0.540	2.046 (0.386-6.442)	0.384
MMF + Tacrolimus	0.684 (0.237-1.978)	0.483	0.748 (0.356-1.571)	0.443
