# Supplementary figures and images for: Clinical outcomes of COVID-19 infection in liver transplant recipients based on vaccination status
Source: Front Transplant. 2025 Jan 9;3:1515964. doi: 10.3389/frtra.2024.1515964 (PMC11754219; doi:10.3389/frtra.2024.1515964)

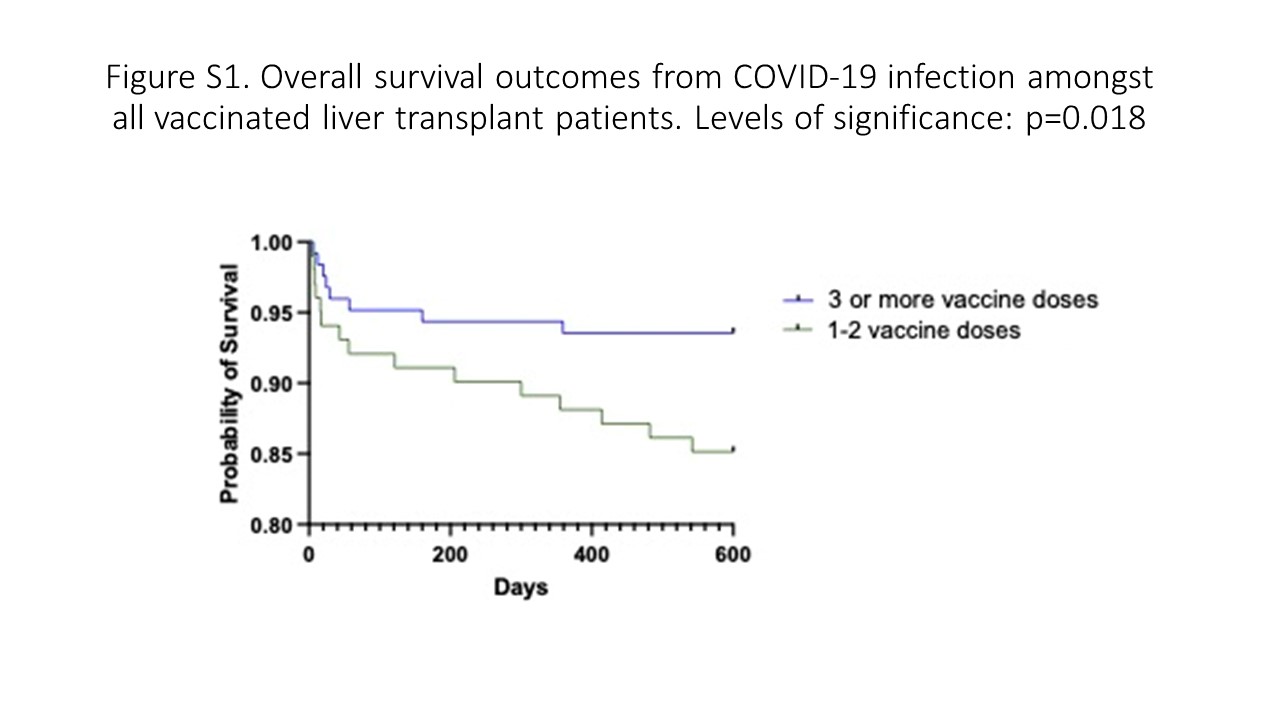

Supplement: Supplementary file 3 [file Image1.jpg]
